# Isolated double-orifice mitral valve: a case report

**DOI:** 10.1186/s12872-015-0168-0

**Published:** 2015-12-18

**Authors:** Philipp Krisai, Bastian Wein, Beat A. Kaufmann

**Affiliations:** Department of Medicine, University Hospital Basel, Petersgraben 4, 4031 Basel, Switzerland; Cardiovascular Research Institute Basel, University Hospital Basel, Basel, Switzerland; Department of Cardiology, University Hospital Basel, Petersgraben 4, 4031 Basel, Switzerland

**Keywords:** Isolated double-orifice mitral valve, Cardiac anomaly, Mitral valve, Valve anomaly

## Abstract

**Background:**

Double-orifice mitral valve is an extremely rare cardiac anomaly possibly originating from insufficient endocardial fusion in embryogenesis. Severe concomitant cardiac anomalies and malfunction of the valve usually lead to an early diagnosis in childhood. Therefore the prevalence of isolated double-orifice mitral valve in adulthood is not known.

**Case presentation:**

We present the case of a 63 years old, female Caucasian patient with isolated double-orifice mitral valve diagnosed in routine echocardiographic evaluation after chemotherapy presenting without clinical symptoms.

**Conclusion:**

Trans-thoracic echocardiography is a suitable modality to diagnose and further assess anatomical and functional properties of the anomaly. In the presence of double-orifice mitral valve concomitant cardiac anomalies and valvular stenosis or regurgitation must be excluded. If an isolated double-orifice mitral valve with no functional abnormalities is present, no further follow-up is necessary.

**Electronic supplementary material:**

The online version of this article (doi:10.1186/s12872-015-0168-0) contains supplementary material, which is available to authorized users.

## Background

Isolated double-orifice mitral valve (DOMV) is an extremely rare congenital anomaly, possibly originating from insufficient embryonic fusion of the endocardial cushions [1, 2], leading to two separate orifices of the mitral valve into the left ventricle. Because of frequent associated anomalies and impaired function [3], the anomaly is usually detected in early childhood. The prevalence and prognostic relevance of an isolated DOMV in adulthood is not known.

## Case presentation

**Fig. 1:**
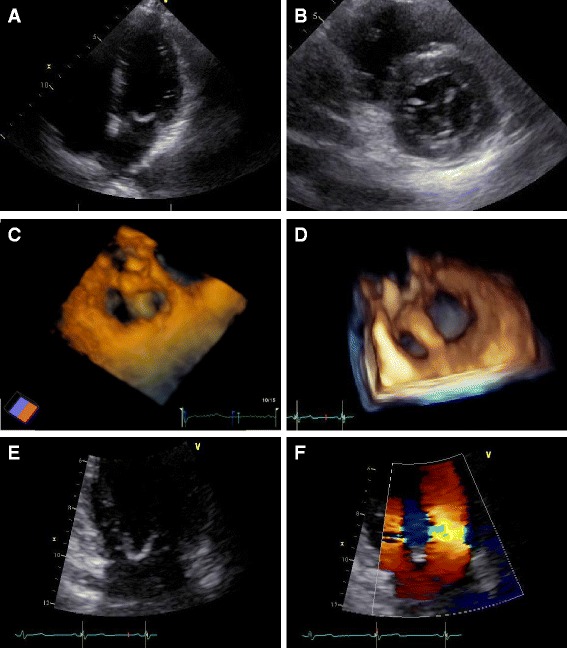
Trans-thoracic echocardiography demonstrating the DOMV in a modified 4-chamber view (**a**), a 2-dimensional short-axis (**b**) and two 3-dimensional images (**c**, **d**) demonstrating the two mitral orifices, and the fibrous bridge separating orifices. Zoomed images (**e**, **f**) demonstrate the separate inflow jets through the orifices.

We present the case of a 63 years old Caucasian female, referred to our echocardiography lab for routine evaluation 5 years after chemotherapy including cyclophosphamid and stem cell transplantation for chronic myeloid leukemia. She was in good general condition and asymptomatic. Physical examination, electrocardiography, prior thoracic X-ray and CT-scan were unremarkable. On trans-thoracic echocardiography (TTE), performed with a Philips iE33, a DOMV could be detected in standard 2-dimensional views without stenosis or regurgitation (Fig. 1a, b, e and f, Additional file 1: Movie 1, Additional file 2: Movie 2, Additional file 3: Movie 3, Additional file 4: Movie 4 and Additional file 6: Movie 6). We classified the DOMV as a complete bridge type with a fibrous bridge separating the valve into two orifices with a slightly larger anterolateral orifice. Each orifice had its own leaflets and subvalvular apparatus, each connected to one papillary muscle resulting in a double parachute appearance in apical views (Fig. 1a and b, Additional file 1: Movie 1, Additional file 2: Movie 2 and Additional file 3: Movie 3). Two separate jets of antegrade flow into the left ventricle were detected with color Doppler flow (Fig. 1e and f, Additional file 5: Movie 5 and Additional file 6: Movie 6). We furthermore evaluated the valve with three dimensional TTE (Fig. 1c and d, Additional file 4: Movie 4), illustrating the relative dimensions of the two orifices. Interestingly, no other congenital cardiac anomaly or structural cardiopathy was found. In the absence of cardiac symptoms in this patient with a DOMV with normal function no further diagnostic or therapeutic steps were necessary. The patient was instructed to return to our clinic if any symptoms should evolve.

## Discussion

First described in 1876 [4], DOMV is an extremely rare congenital anomaly, classified into three categories: complete bridge (15 %), incomplete bridge and hole type (85 %) [1]. DOMV is believed to originate from insufficient fusion of endocardial cushion, which can also be responsible for associated anomalies [2]. In a large autopsy series of patients with congenital heart disease, 1 % had a DOMV, which in the majority of cases was associated with other cardiac anomalies (atrioventricular septal defect, coarctation of the aorta, patent ductus arteriosus, interrupted aortic arch) or mitral regurgitation [3]. Because of the associated anomalies, DOMV is usually detected in early childhood but is an exceedingly rare diagnosis in adults, and thus the incidence and prognostic relevance of an isolated DOMV detected in adulthood is unknown.

As an isolated DOMV without functional abnormalities of the mitral valve does not necessarily cause clinical symptoms it is prone to be missed. Our case demonstrates a rare case of isolated DOMV without apparent clinical implications diagnosed very late at 63 years of age, underlining the need for comprehensive and accurate TTE in patients referred for “routine” exams. Furthermore we show that TTE is an appropriate diagnostic tool to not only diagnose, but also evaluate the anomaly in 2D and 3D imaging modalities.

## Conclusions

Echocardiography is a suitable modality for diagnosing DOMV and offers the possibility of comprehensive anatomical and functional assessment. In the presence of DOMV concomitant cardiac anomalies and valvular stenosis or regurgitation must be excluded. If an isolated DOMV with no functional abnormalities is present, no further follow-up is necessary.

### Consent

Written informed consent was obtained from the patient for publication of this Case report and any accompanying images. A copy of the written consent is available for review by the Editor-in-Chief of this journal.

## Additional files

Additional file 1: Movie 1. Trans-thoracic 2-dimensional short axis view. (MP4 687 kb) Additional file 2: Movie 2. Trans-thoracic 2-dimensional modified 4-chamber view. (MP4 598 kb) Additional file 3: Movie 3. Trans-thoracic 2-dimensional 2-chamber view. (MP4 676 kb) Additional file 4: Movie 4. Trans-thoracic 3-dimensional view. (MP4 571 kb) Additional file 5: Movie 5. Enlarged trans-thoracic 2-dimensional 2-chamber view. (MP4 549 kb) Additional file 6: Movie 6. Enlarged trans-thoracic 2-dimensional 2-chamber view with colour Doppler flow. (MP4 738 kb)

## Abbreviations

DOMV: double-orifice mitral valve
TTE: trans-thoracic echocardiography
